# Appetite control and gastrointestinal hormonal behavior (CCK, GLP-1, PYY 1–36) following low doses of a whey protein-rich nutraceutic

**DOI:** 10.1007/s12349-013-0121-7

**Published:** 2013-02-05

**Authors:** Samir Giuseppe Sukkar, Alberto Vaccaro, Giovanni Battista Ravera, Claudia Borrini, Raffaella Gradaschi, Anna Massa Sacchi-Nemours, Renzo Cordera, Gabriella Andraghetti

**Affiliations:** 1 Clinical Nutrition Unit, IRCCS Azienda Ospedaliera Universitaria San Martino-IST di Genova, Genova, Italy; 2 Bio Statistical Institute, Health Science Department (DISSAL), Genoa University, Largo R. Benzi 2, 16122 Genova, Italy; 3 Department of Endocrinology and Metabolism, IRCCS Azienda Ospedaliera Universitaria San Martino-IST di Genova, Genova, Italy

**Keywords:** Whey protein, Appetite suppressant, GI hormones, CCK, GLP-1, PYY, Obesity

## Abstract

Whey proteins represent the most satiating nutrients. In particular, their effects are due to enterohormonal changes (CCK, GLP-1 and PYY 1–36) observed after their exclusive ingestion. Glucomannan has important satiety property due to volume increase following gelification. The aim of the study is the evaluation of subjective rate of hunger and enterohormone concentrations (CCK, GLP-1, PYY 1–36) following oral loading of a mixture containing WP (8 g) or casein (8 g) plus glucomannan (1 g) (Colordiet^®^, Inpha DUEMILA Srl Lecco, Italy). The study was conducted as a double-blind crossover with five healthy volunteers (BMI 22–26 kg/m^2^ aging 18–65 years) in acute and a wash-out period of 1 week between the first and the second evaluation. From the analysis of the data, we observe that the load with WP induces a significant decrease in the desire to eat after 90 min (*P* < 0.0446) when compared with casein. As far as plasma hormones are concerned, there was a significant increase only in GLP-1 at 90 min after WP (*P* *<* 0.00166) and 180 min after casein (T0 vs. T180 *P* = 0.000129). There is a significant correlation between the increase in GLP-1 and decrease of desire to eat (*R* = −0.93). There is a tendency to the increasing of CCK after 90 min, which is not significant (*P* = 0.091). These results could be due to (a) the low number of cases or (b) the low dose of protein used. The present study suggests that a mixture of WP plus glucomannan exerts a decrease in the desire to eat which is correlated to enterohormonal modification (GLP-1 increase) despite the low content of protein (8 g) and the presence of glucomannan, which could reduce the fast absorption of WP in relation to the net forming during the gelification of the gastric environment.

## Introduction

Recent studies in animals and humans have shown that whey proteins have anorexigenic properties that may be a potential weapon for overweight management. In particular, the anorectic effect of whey proteins (WP) is stronger than that induced by casein, soy proteins and ovalbumin [1].

Whey proteins may reduce caloric intake as a result of various physiological mechanisms. Casein macropeptide (CMP), which is the precursor protein of glyco macropeptide (GMP), is a bioproduct of rennin (chymosin) and a component of whey (1.2–1.5 g/L). Over 20 years ago, it was shown that GMP can affect gastrointestinal function through the inhibition of gastric secretion [2], and that this action could be mediated by peptide hormones, such as cholecystokinin (CCK) [3, 4], which is a potent activator of satiety signals. The CCK-like activity of GMP was then confirmed by subsequent studies [5, 6]. Its satiating power and weight control action are extremely variable and depend on the physical and chemical properties determining its digestion and absorption with the subsequent release of amino acids. Circulating postprandial amino-acid (AA) levels are amongst the main factors that influence satiety.

There is a wealth of evidence that whey protein effect on satiety and food intake is mediated by the release of satiety-inducing hormones. More than 20 different regulatory peptide hormones are released in the gastrointestinal system. Many of them are involved in regulating food intake and are sensitive to content and composition of intestinal nutrients [9]. A number of them respond after protein consumption and this may account for food intake suppression after milk protein ingestion.

CCK, GLP-1(glucagon-like peptide-1), GIP (Gastric inhibitory polypeptide), PYY (peptide YY), and ghrelin in particular, play a key role. Insulin is involved in the regulation of food intake, both in the short and the long term. Insulin release is stimulated by whey protein ingestion that affects the glycemic response and is closely associated with short-term satiety and decreased food intake [7]. Insulin response has been shown to have a closer correlation with short-term satiety and food intake than do gut hormones.

Anyways, the satiating effects of WP have been proven only when they are administered in a single-dose high load (20–40 g).

This could lead to an protein overload if we consider the recommended dietary allowance (RDA) when the rest of daily food intake is concerned.

Therefore, it could be very interesting to evaluate whether WP lower dosage also determines a similar satiating effect.

The aim of this study was to evaluate the satiety-inducing effect and the effect on satiety-associated gut hormones (CCK, GLP-1, PYY 1–36) of an oral administration of low-load whey proteins versus casein (8 g/dose).

## Materials and methods

A double-blind crossover study has been conducted employing five healthy volunteer subjects [BMI (body mass index) between 22 and 26 kg/mq, aged 25–55 years].

In acute, five samples were performed for the first and the second arm of the study with 1-week washout between the two doses.

Patients excluded from the study were those with the following contraindications:

- eating disorders, particularly binge eating disorder (BED), bulimia;
- known or suspected pregnancy;
- depression and/or obsessive–compulsive disorder treated with serotoninergic drugs;
- intake of appetite suppressor drugs;
- inhibiting pancreatic lipase;
- renal failure.

After informed consent has been obtained, each subject underwent the following baseline evaluations:

1. anamnesis and physical examination;
2. anthropometric measures: body weight with calculation of BMI and waist circumference, and
3. assessment of average daily calorie intake by food diary

The study was conducted after an overnight fast and the samples were carried out as follows:

1. The satiating capacity was evaluated by means of Haber’s scale, an analog visual scale from −10 (representing extreme hunger: painfully hungry) to +10 (representing extreme satiety: full to nausea) [8]. In particular, the subjects describe the level of agreement with respect to hunger or satiety by pointing to an appropriate place along the graduated visual scale. Assessment of tolerability (adverse events monitoring and reference), which has been transformed in a scale from 1 to 20 for an easy report.
2. Detection of side effects.
3. Five samples were made for each study arm (T0: 0′; T1: 15′; T2: 30′; T3: 90′; T4: 180′).
4. CCK, GLP-1 and PYY were assayed in duplicate using radioimmunoassay (RIA kit—Phoenix Pharmaceuticals, Inc. (Burlingame, California, USA), according to the instructions provided.

The procedure contemplates:

*Plasma collection* the blood was collected into tubes containing EDTA (Greiner VACUETTE^®^ K3E K3EDTA, 4 mL, lavender/black, 13 × 75 mm), ridged and stirred gently to prevent coagulation. Then it was transferred into tubes containing aprotinin (0.6 TIU/mL of blood), stirred gently several times to inhibit the activity of proteinase and centrifuged at 1,600×*g* for 15 min at 4 °C. The plasma was stored at −70 °C.

*Plasma extraction* the plasma was acidified and centrifuged at 10,000×*g* for 20 min at 4 °C Then it was loaded onto columns containing 200 mg of Sep Column C18, equilibrated with Buffer B (Buffer B: 60 % acetonitrile in 1 % TFA) and A [Buffer A: 1 % trifluoroacetic (TFA)]. (Phoenix Pharmaceuticals).

The eluate was collected in tubes of polystyrene, concentrated, lyophilized and then reconstituted with RIA buffer.

### Product features

The composition of the product (containing whey proteins) (Colordiet^®^, INPHA DUEMILA S.r.l., via Elettrochimica n.37, Lecco, Italy) was:

- proteins of the whey concentrate 80 % 10,000.0 mg
- starchlite white bean seeds e.g. aaiu 3,000 (3,000 alpha amylase inhibiting unit) 500.0 mg
- glucomannan 1,000.0 mg
- excipients, sweeteners, flavoring or similar tropical

The composition of the product (containing casein) was:

- Casein of milk concentrated 80 % 10,000.0 mg
- starchlite white bean seeds e.g. aaiu 3,000 (3,000 alpha amylase inhibiting unit) 500.0 mg
- glucomannan 1,000.0 mg
- excipients, sweeteners, flavoring or similar tropical

### Statistical evaluation

Five healthy subjects were enrolled in this crossover study.

All data were tabulated and subjected to appropriate statistical analysis by the biostatistics SPSS package.

Effectiveness and tolerability regarding the subjects who completed the study (drop-outs were replaced) were measured. Student’s *t* tests were used for parametric data and the Wilcoxon’s test was relied on for non-parametric tests.

The non-parametric Friedman test was applied to evaluate the differences in satiation feeling (Haber’s score). If significant differences were identified, all the possible pairwise comparisons were investigated with non-parametric Wilcoxon’s test for paired data, adjusting the reference, the *P* value (0.05), according to Bonferroni’s method.

The efficacy on the feeling of satiety was assessed by the net change from the baseline at the end of follow-up. Unpaired *t* test (with Satterthwaite’s correction for degrees of freedom) or the analogous non-parametric Wilcoxon–Mann–Whitney’s test (MW) was applied. A value of *P* < 0.05 was considered significant (two-side).

## Results

Analysis of the data showed that the WP administration induced a significant decrease in appetite after 90 min (*P* < 0.05) compared to casein (Table 1).

**Table 1:** Appetite and enterohormonal profile following whey proteins

Time (min.)	0′	15′	30′	90′	180′
Appetite	15.0 ± 4.3	12.6 ± 4.3	12.6 ± 4.2	12.2 ± 4.0*	13.2 ± 4.8
GLP-1 pmol/l	6.16 ± 1.78	6.80 ± 1.61	6.94 ± 1.7	7.11 ± 1.08**	6.77 ± 2.09
CCK ng/mL	1.1 ± 0.1	1.17 ± 0.18	1.17 ± 0.17	1.3 ± 0.25	1.16 ± 0.16
PYY pg/mL	19.7 ± 0.3	19.4 ± 0.5	19.6 ± 0.3	19.7 ± 0.5	19.7 ± 0.4

*CCK* NS, *PYY* NS

* Appetite: T0 vs. T90 *P* = 0.0446

** GLP-1: T0 vs. T90 *P* = 0.0166

Plasma gut hormone concentration increased significantly only for GLP-1 (*P* < 0.05) after 90 min in WP group, while after 180 min (*P* < 0.05) in the casein group (Tables 1, 2). There is a significant correlation between GLP-1 and decrease in appetite in WP (*R* = −0.93). CCK concentration tends to increase after 90 min even though it does not attain statistical significance (*P* = 0.091).

**Table 2:** Appetite and enterohormonal profile following casein

Time (min.)	0′	15′	30′	90′	180′
Appetite	11.4 ± 7.8	9.6 ± 6.4	7.7 ± 4.7	9.4 ± 5.5	11.8 ± 6.8
GLP-1	7.0 ± 1.1*	7.1 ± 1.2	6.74 ± 1.1	7.2 ± 1.2	7.2 ± 1.2*
CCK	1.4 ± 0.1	1.3 ± 0.2	1.3 ± 0.1	1.4 ± 0.1	1.4 ± 0.1
PYY pg/mL	19.7 ± 0.3	19.5 ± 0.3	19.7 ± 0.4	19.8 ± 0.5	19.9 ± 0.6

*Appetite* NS, *CCK* NS, *PYY* NS

* GLP-1: T0 vs. T180 *P* = 0.0129

## Discussion and conclusions

Since, glucomannan cannot affect gut hormone response [9–13] and similarly, phaseolamin does not affect gut hormone-mediated appetite modulation because it blocks amylase in the digestion of complex carbohydrates (starch), the discussion will focus on the different protein compositions of the supplement [14–17].

It is well known that, compared to a standard diet, a high-protein diet increases satiety, postprandial thermogenesis (by decreasing energy efficiency in futile cycles), basal metabolism, protein balance, and fat oxidation [17]. In addition, high biological value proteins have a greater effect on energy expenditure than the others. Finally, a protein-rich diet seems to have a positive effect on bone mineralization and to cause a reduction of fractures due to osteoporosis [18]. Physiologically, a high-protein diet produces a prolonged feeling of satiety by stimulating the secretion of anorectic hormones [19].

Despite these interesting premises, a highly hyper protein diet (>2 g/kg/day) can cause nitrogen overload on the kidney due to sulfur amino acids, therefore, caution should be taken in considering this approach in patients with or at risk of renal disease and those with metabolic syndrome, [1]. This is why in recent years, more attention has been focused on evaluating the anorectic components of proteins to check the difference in their compositions so that they may be used in a more rational and modular manner.

Veldhorst et al. [20] noted that in a diet where 10 % of energy was provided by proteins, WP showed a greater satiating effect than caseins and soy proteins. When dietary protein provided 25 % of energy, differences in satiety induced by the three different types of proteins were no longer observed, although the WP induced a greater hormonal stimulation. The authors concluded that the differences observed were the result of the different amounts of amino acids, some of which were present in greater concentrations, beyond the threshold for activation of anorectic gut hormone secretion.

Recent studies in animals and humans have shown that WP have anorexigenic properties that may be a potential weapon for overweight management [20–23]. In particular, studies by Sukkar and others [21, 22] have shown that WPs are able to reduce food intake and consequently gain weight both in Wistar and in obesity-prone Zucker rats.

Whey proteins may reduce caloric intake as a result of various physiological mechanisms. CMP, which is the precursor protein of GMP, a bioproduct of rennin (chymosin) and a component of whey (1.2–1.5 g/L) may have anorectic activity. Over 20 years ago, it was shown that GMP can affect gastrointestinal function through the inhibition of gastric secretion [24], and that this action could be mediated by peptide hormones, such as CCK [25, 26], a potent activator of satiety signals. The CCK-like activity of GMP was confirmed by subsequent studies [27, 28].

Studies by Veldhorst et al. [29] confirmed that, being a WP fraction, GMP reduces energy intake because of its greater content in amino acids: serine, threonine, alanine, isoleucine, and α-aminobutyric acid, regardless of the remaining WP components. Whey proteins were found to determine a maximum satiety response in that once a certain anorectic hormone concentration is reached, the effect on satiety plateaus irrespective of the progressive increase in anorectic hormones due to additional whey protein intake [29].

The control of satiety by WP is also determined by a second mechanism related to their physical and chemical properties. These properties bring about different digestion and absorption modalities resulting in differing degrees of amino-acid release, with circulating postprandial AA levels acting as one of the main factors that influence satiety.

Proteins may be “fast” or “slow” depending on their different digestion and absorption times and on their contribution to protein synthesis and postprandial plasma AA concentrations [30]. After ingestion, WP rapidly pass through the stomach and reach the jejunum as intact proteins. By contrast, casein undergoes substantial digestion in the stomach due to the strongly acid environment and coagulates forming lumps, passing more slowly through the small intestine. In the small intestine, whey protein is hydrolyzed more slowly than the other proteins, and digested and absorbed within a wider tract [31].

Based on the plasma amino-acid concentrations observed after an oral load, whey digestion and absorption of its amino acids were found to be clearly more rapid than casein. Similarly, mice and rats fed with whey reduce their food intake 30 min after administration than when fed with casein [21, 32].

Whey proteins are considered to be the highest quality proteins with the best biological value [33]. The protein efficiency ratio (PER), which reflects the weight gain in laboratory animals per gram of protein taken after 4 weeks, is very high (3.2) and greater than casein (2.6).This feature may be one of the factors that determine their satiating power since, compared to other proteins, whey proteins contain a higher concentration of branched-chain amino acids, especially l-leucine. Sweet and dry wheys contain 10.3 and 10.5 % leucine, respectively [34]. Leucine acts on an mTOR-dependent kinase that is non-insulin dependent, promotes anabolism of muscle tissue, maintains stable glucose levels, and lowers insulin during energy restriction [35]. Leucine crosses the blood–brain barrier more rapidly than other amino acids [36], and its important role in hypothalamic regulation of food intake has been demonstrated in the recent studies [36].

Direct intracerebroventricular injection of amino-acid mixtures (RPMI 1640) and leucine alone (1 g) inhibits food intake for 24 h, indicating that an increase in brain amino-acid concentrations is sufficient to suppress food intake. This effect is related to increased mRNA Agrp (Agouti-related protein) levels in hypothalamic GT1-7 cells exposed to low-amino-acid concentrations for 16 h, and is attenuated when leucine is removed from the cell culture [37]. It may be mediated by metabolic-sensing neurons [38, 39] via an mTOR-dependent mechanism [40], in which an increase in the brain amino-acid concentration leads to appetite inhibition [41].

Increasing evidence has shown that the effect of whey proteins on satiety and food intake is mediated by the effect of satiety-inducing hormone release [7, 42].

Cholecystokinin is known as a satiety hormone [7], in rats, CCK and its receptor subtype A are involved in the suppression of food intake induced by proteins [43, 44]. In humans, proteins and fats in the diet are the main stimulators of CCK secretion [45], and digestion of proteins is necessary for the release of CCK [46]. Milk proteins increase CCK plasma concentration, with an initial peak after 15–20 min, following, which there is a fall and a subsequent increase after about 90 min [23, 47].

Whey increased CCK concentration to a greater extent than did casein in a study by Hall [23] but not in one by Bowen [47]. This could be due to differences in the GMP content of the wheys used in the investigations.

Glucagon-like peptide-1 also appears to play a role in protein-induced satiety [47]. Both carbohydrates and fats are potent stimulators of GLP-1 [48], but milk proteins stimulate the release of GLP-1 regardless of the presence or absence of carbohydrates and fats [48].

Whey appears to have a more potent action and its secretagogue effect can be increased in the presence of other micronutrients.

A high-protein breakfast (58 % of total energy), consisting mainly of whey protein-enriched dietetic products, increases GLP-1 concentrations after 3 h compared to a high-carbohydrate breakfast with yoghurt (mainly casein) [49].

In human studies, a whey protein load (50 g) administered with 200 kcal from fats and carbohydrates caused greater GLP-1 plasma concentrations than casein, again after 3 h [23].

Evidence of the indirect role of GLP-1 on milk protein-induced satiety was provided by studies on rats showing that exendin-4 (Ex-4), a GLP-1 receptor agonist, interacts with milk proteins in suppressing food intake [50, 51]. This effect was observed both when the proteins were administered intact, partially hydrolyzed or in the form of free amino acids [51].

The glucose-dependent insulinotropic polypeptide, GIP, is released by K cells in the duodenum after food intake, and may have an important role in the development of obesity, since GIP receptor knockout mice were observed to be resistant to obesity even if fed a high-fat diet [42]. Recent studies have shown that a whey-containing beverage significantly increased GIP response (to 80 %) in healthy subjects, while mixtures of branched-chain amino acids did not have the same effect [52].

It is possible that bioactive peptides present in whey or formed during digestion are the main stimulators of GIP secretion [52]. Branched-chain amino-acid mixtures do not stimulate gut hormone (GIP and GLP-1) response, whereas whey protein drinks produce an increased response, suggesting that the action of whey proteins is not simply related to the amino-acid content but it is also due to the action of peptics [52].

The peptide YY is a gut hormone secreted by intestinal L cells and is present throughout the intestinal tract, with a higher concentration in the distal ileum [42]. PYY is secreted postprandially proportional to caloric intake and depends on macronutrient composition [53]. PYY plasma concentration increases after the intragastric administration of whey proteins or whey peptides hydrolysates in healthy subjects, and it is not related to the degree of protein fractionation [52]. No comparisons between the effect of whey and that of other proteins have been reported.

Ghrelin is a digestive orexigenic hormone [42] released into the circulation by the stomach; its concentrations generally reach a peak before meals, while its action is suppressed by food intake [54]. Like other digestive hormones, ghrelin plasma response depends on the macronutrient composition of the meal.

All three classes of macronutrients can suppress plasma ghrelin, but with different levels of efficiency [54].

Loads of milk protein, casein, whey or GMP in rats all produced a similar reduction of ghrelin plasma concentrations after 30 min compared to placebo (water alone) [32].

In humans, whey proteins and calcium caseinate suppress ghrelin concentrations similarly to lactose and more than glucose after 3 h. This effect was correlated with a further reduction in subsequent energy intake [48].

Milk proteins and particularly the whey protein fraction have bioactive properties (hormones, growth factors, cytokines, antioxidants and stimulation of protein synthesis), which determine physiological regulations. Whey proteins also have an antioxidant effect that could be useful in controlling the oxidative stress occurring in the metabolic syndrome. They increase glutathione (GSH) synthesis and have been shown to boost immunity by increasing lymphocyte and splenic GSH synthesis [21, 55–64].

Recently, Pall et al. [65] have shown that total and LDL cholesterol reduced significantly after a 12-week treatment with whey proteins versus casein, in 70 obese human subjects (BMI = 1.3 ± 0.8 kg/m^2^)

In sum, analysis of the findings reported revealed that a whey protein load induced a significant decrease in eating desire after 90 min (*P* < 0.05), compared to casein. As for the gut hormone plasma concentrations, only GLP-1 increased significantly (*P* < 0.05) 90 min after whey protein administration and 180 min after casein administration (*P* < 0.05) and there was a significant correlation between GLP-1 and decreased eating desire (*R* = −0.93). CCK concentrations also tended to increase after 90 min, but this is not statistically significant (*P* = 0.091) for whey proteins. PYY displayed no significant changes after either whey protein or casein loads.

These results may be due to:

1. the small number of cases studied
2. the small amount of proteins used.

In fact, other experiments have actually shown a significant increase in CCK and PYY after administration of 20–58 g of whey proteins.

With regard to PYY, the findings reported are not consistent with the data described in the literature for the same reasons itemized for CCK. Given the small amount of whey proteins used, the proteins may not have reached the site where there is a greater PYY secretion as they are absorbed in the duodenum and jejunum, while the PYY-secreting cells are mainly localized in the distal ileum [66].

In conclusion, this study suggests that a mixture of whey proteins and glucomannan reduces eating desire and that this effect is related both to the changes in gut hormone levels (GLP-1) despite the low protein content (10 g), and to the presence of glucomannan that forms a “net” after gelling in the acid milieu of the stomach.

It is interesting to note that the result was achieved despite the low protein content (10 g), which is compatible with a clinical use of product. Elsewhere in the literature mixtures containing 50 g of whey proteins had been used [10].

In light of these results, we suggest that dietary supplementation with whey proteins might be employed as a treatment for appetite control. Whey proteins present the added value of being a source of branched-chain amino acids and leucine that are particularly appropriated for lean body mass maintenance in the course of low-calorie diets.

Future studies on larger populations are called for further investigations to analyze the effects both on appetite and hormones (CCK, GLP-1, PYY 1–36), and varying the amounts of whey proteins versus caseins.
